# Latent class bivariate model for the meta-analysis of diagnostic test accuracy studies

**DOI:** 10.1186/1471-2288-14-88

**Published:** 2014-07-11

**Authors:** Paolo Eusebi, Johannes B Reitsma, Jeroen K Vermunt

**Affiliations:** 1 Department of Epidemiology, Regional Health Authority of Umbria, Via Mario Angeloni, 61, 06124 Perugia, Italy; 2 Neurologic Clinic, Department of Medicine, University of Perugia, Perugia, Italy; 3 Julius Center for Health Sciences and Primary Care, University Medical Center, Utrecht, The Netherlands; 4 Department of Methodology and Statistics, Tilburg University, Tilburg, The Netherlands

**Keywords:** Meta-analysis, Meta-regression, Bivariate model, Latent class model

## Abstract

**Background:**

Several types of statistical methods are currently available for the meta-analysis of studies on diagnostic test accuracy. One of these methods is the Bivariate Model which involves a simultaneous analysis of the sensitivity and specificity from a set of studies. In this paper, we review the characteristics of the Bivariate Model and demonstrate how it can be extended with a discrete latent variable. The resulting clustering of studies yields additional insight into the accuracy of the test of interest.

**Methods:**

A Latent Class Bivariate Model is proposed. This model captures the between-study variability in sensitivity and specificity by assuming that studies belong to one of a small number of latent classes. This yields both an easier to interpret and a more precise description of the heterogeneity between studies. Latent classes may not only differ with respect to the average sensitivity and specificity, but also with respect to the correlation between sensitivity and specificity.

**Results:**

The Latent Class Bivariate Model identifies clusters of studies with their own estimates of sensitivity and specificity. Our simulation study demonstrated excellent parameter recovery and good performance of the model selection statistics typically used in latent class analysis. Application in a real data example on coronary artery disease showed that the inclusion of latent classes yields interesting additional information.

**Conclusions:**

Our proposed new meta-analysis method can lead to a better fit of the data set of interest, less biased estimates and more reliable confidence intervals for sensitivities and specificities. But even more important, it may serve as an exploratory tool for subsequent sub-group meta-analyses.

## Background

There is an increasing interest in meta-analyses of data from diagnostic accuracy studies [1-4]. Typically, the data from each of the primary studies are summarized in a 2-by-2 table cross-tabulating the dichotomized test result against the true disease status, from which familiar measures such as sensitivity and specificity can be derived [5]. Several statistical methods for meta-analysis of data from diagnostic test accuracy studies have been proposed [6-13]. Generally, we expect that such data show a negative correlation between sensitivity and specificity because of explicit or implicit variations in test-thresholds [1,7], as well as contain a certain amount of heterogeneity [14]. Our research is motivated by the need to explore and explain sources of heterogeneity in systematic reviews of diagnostic tests in a more careful manner. In fact, methods for the meta-analysis of sensitivity and specificity are still an active field of research and debate. One frequently used method involves generating a ROC curve using simple linear regression [6,7]. However, the assumptions of the underlying linear regression model are not always met, and as a consequence the produced statistics, in particular standard errors and p-values, may be invalid. There is also uncertainty as to the most appropriate weighting of studies to be used in the regression analysis [15]. Rutter and Gatsonis [9] suggested that in the presence of a substantial amount of heterogeneity, the results of meta-analyses should be presented as Summary ROC curves.

Reitsma and others [12] proposed the direct analysis of sensitivity and specificity estimates using a bivariate model BM, which yields a rigorous method for the meta-analysis of data on diagnostic test accuracy, in particular when the studies are selected based on a common threshold [16]. Chu and Cole [17] extended this bivariate normal model by describing the within-study variability with a binomial distribution rather than with a normal approximation of transformed observed sensitivities and specificities. Though the BM may work well with the normal approximation, Hamza [18] and others suggested that the binomial distribution is to be preferred, especially when only few studies with a small size are available. An additional advantage of the binomial approach is that it does not require a continuity correction. When using a bivariate generalized linear mixed model to jointly model the sensitivities and specificities, different monotone link functions can be implemented, such as logit, probit, and complementary log-log transformations [19]. Chu and others [20] also discussed a trivariate nonlinear random-effects model for jointly modeling disease prevalence, sensitivity and specificity, as well as an alternative parameterization for jointly modeling prevalence and predictive values.

Bayesian modeling approach to BM are gaining popularity, by allowing the structural distribution of the random effects to depend on multiple sources of variability and providing the predictive posterior distributions for sensitivity and specificity [21]. In order to avoid the Markov chain Monte Carlo sampling also a deterministic Bayesian approach using integrated nested Laplace approximations have been proposed [22]. BM can be seen within a unified framework which includes also the Hierarchical Summary ROC model [23]. Arends and others [24] showed that the bivariate random-effects approach not only extends the Summary ROC approach but also provides a unifying framework for other approaches. Rücker and Schumacher [13] proposed an alternative approach for defining a Summary ROC curve based on a weighted Youden index.

The Latent Class Model has been introduced in the literature as a tool for evaluating the accuracy of a new test when there is no gold standard against which to compare it [25,26]. A probabilistic model is assumed for the relationship between the new diagnostic test, one or more imperfect reference tests, and the unobserved, or latent, disease status. This provides estimates of the sensitivity and specificity of the new diagnostic test. This application of latent class analysis has received considerable attention in the context of primary (individual) diagnostic accuracy studies, but its use in meta-analysis is rare [27].

In this paper we propose using a latent class approach for a different purpose. More specifically, we use it as a tool for clustering the studies involved in the meta-analysis [28]. For this purpose, the BM based on using a binomial distribution is expanded to include a discrete latent variable. The resulting Latent Class Bivariate Model (LCBM) allows obtaining more reliable estimates of sensitivity and specificity, as well as the estimation of a different between-study correlation between sensitivity and specificity per latent class. While this correlation is usually assumed to be negative, this is not always correct in real-data applications, probably due to varying accuracy levels and differences in test performance. In a simulation study, the LCBM is compared to the standard BM in terms of bias, power, and confidence. Moreover, it is applied to a well-known dataset on the diagnostic performance of multislice computed tomography and magnetic resonance imaging for the diagnosis of coronary artery disease [16,29].

The remaining of the article is organized as follows. We first describe the BM and the LCBM, as well as discuss computational issues and the setup of the simulation study. Then, attention is paid to the results of the simulation study and the application of the LCBM to the coronary artery disease data. Next, we discuss the implications of our study and present some conclusions. Software code for estimating the LCBM is provided as Additional files.

## Methods

### The bivariate model

The BM is based on an approach to meta-analysis introduced by Van Houwelingen and others [8], which has also been applied to the meta-analysis of diagnostic accuracy studies [12]. Let *x*_1*i*_ and *n*_1*i*_ be the number of subjects with a positive test result and the total number of subjects with the disease in study *i*, respectively, and *x*_0*i*_ and *n*_0*i*_, analogously, be the number of subjects with a negative test result and the total number of subjects without the disease. Then, the observed sensitivity and specificity is *x*_1*i*_/*n*_1*i*_ and *x*_0*i*_/*n*_0*i*_. The corresponding true values are denoted by *η*_*i*_ and *ξ*_*i*_, respectively. The BM can be specified as follow [17]:

(1) $$\begin{array}{l} x_{1i}∼\text{binomial}(\eta_{i},n_{1i})\\ x_{0i}∼\text{binomial}(\xi_{i},n_{0i}) \end{array}$$

(2) $$\begin{array}{l} \text{logit}(\eta_{i})=X_{i}\alpha +\mu_{i}\\ \text{logit}(\xi_{i})=W_{i}\beta +\nu_{i} \end{array}$$

(3) $$\left(\begin{array}{l} \mu_{i}\\ \nu_{i} \end{array}\right)∼N\left(\begin{array}{c} 0\\ 0 \end{array}\right)\;\left(\begin{array}{cc} \sigma_{\eta }^{2} & \rho \sigma_{\eta }\sigma_{\xi }\\ \rho \sigma_{\eta }\sigma_{\xi } & \sigma_{\xi }^{2} \end{array}\right).$$

Here, **X**_*i*_ and **W**_*i*_ are (possibly overlapping) vectors of covariates related to sensitivity and specificity, $\sigma_{\eta }^{2}$ and $\sigma_{\xi }^{2}$ are the between-study variances of sensitivity and specificity, and *ρ* is their correlation. Thus, the parameters of the covariance matrix quantify the amount of heterogeneity present across studies together with how strongly the sensitivity and specificity of a study are related.

The hierarchical model specified by (1), (2) and (3) can be fitted using the generalized linear mixed model procedures in several standard statistical packages. Other kinds of link functions can be used instead of the logit one [19]. In simulation experiments, it has been shown that, in general, it is better to work with such a binomial model rather than with a normal model for transformed observed proportions [18].

### The latent class bivariate model

In the BM, between-study heterogeneity modelled with covariates (2) and random effects (3). We extend the modeling of the between-study heterogeneity by assuming that each study belongs to one of *K* latent classes [28]. This implies expanding the BM model with a discrete latent variable with categories denoted by *c*. The basic structure of the resulting LCBM is:

(4) $$P(x_{1i},x_{0i})=\overset{K}{\underset{c=1}{\sum }}P(c)P(x_{1i},x_{0i}|c),$$

where *P*(*c*) is the probability of a study to belong to latent class *c*, and *P*(*x*_1*i*_,*x*_0*i*_|*c*) is the joint sensitivity and specificity distribution within latent class *c*. For *P*(*x*_1*i*_,*x*_0*i*_|*c*) we specify a BM model with all parameters (including the variances and the correlation) varying across classes. More specifically, in the LCBM the binomial probabilities have the following form:

(5) $$\begin{array}{l} x_{1i}∼\text{binomial}(\eta_{i|c},n_{1i})\\ x_{0i}∼\text{binomial}(\xi_{i|c},n_{0i}) \end{array}$$

(6) $$\begin{array}{l} \text{logit}(\eta_{i|c})=X_{i}\alpha +\mu_{i|c}\\ \text{logit}(\xi_{i|c})=W_{i}\beta +\nu_{i|c}. \end{array}$$

True sensitivities and specificities *η*_*i*|*c*_ and *ξ*_*i*|*c*_ conditional on study *i* belonging to class *c* are assumed to have a bivariate normal distribution with parameters that vary across classes:

(7) $$\left(\begin{array}{l} \mu_{i|c}\\ \nu_{i|c} \end{array}\right)∼N\left(\begin{array}{l} 0\\ 0 \end{array}\right)\;\left(\begin{array}{cc} \sigma_{\eta |c}^{2} & \rho_{c}\sigma_{\eta |c}\sigma_{\xi |c}\\ \rho_{c}\sigma_{\eta |c}\sigma_{\xi |c} & \sigma_{\xi |c}^{2} \end{array}\right).$$

where $\sigma_{\eta |c}^{2}$, $\sigma_{\xi |c}^{2}$ are the class-specific between-study variances and *ρ*_*c*_ is the class-specific between-study correlation. Finally, sensitivity and specificity are assumed to be mutually independent given the latent class memberships and the random effects. This yields the following expression for *P*(*x*_1*i*_,*x*_0*i*_|*c*):

(8) $$\;P(x_{1i},x_{0i}|c)=\int_{\mu }\int_{\nu }P(x_{1i}|c,\mu )P(x_{0i}|c,\nu )f(\mu ,\nu |c)\mathrm{d\mu d\nu .}$$

An additional extension involves the inclusion of covariates **Z**_*i*_ to predict the latent class membership in the LCBM, which yields:

(9) $$P(x_{1i},x_{0i}|Z_{i})=\overset{K}{\underset{c=1}{\sum }}P(c|Z_{i})P(x_{1i},x_{0i}|c).$$

Here, *P*(*c*|**Z**_*i*_) is the probability that a study belongs to latent class *c* given the covariate set **Z**_*i*_ and where, as above, *P*(*x*_1*i*_,*x*_0*i*_|*c*) denotes the class-specific sensitivity and specificity distribution. As can be seen, covariates **Z**_*i*_ affect the latent classes but have no direct effects either on the true sensitivity and specificity or the random effects.

The values of the latent class variable given a study’s covariate values is assumed to come from a multinomial distribution. The multinomial probability *P*(*c*|**Z**_*i*_) is typically parameterized as follows:

(10) $$P(c|Z_{i})=\frac{\text{exp}(\gamma_{c|Z_{i}})}{\overset{K}{\underset{c^{\prime }=1}{\sum }}\text{exp}(\gamma_{c|Z_{i}})},$$

with

(11) $$\gamma_{c|Z_{i}}=\delta_{0}+\overset{P}{\underset{p=1}{\sum }}\delta_{p}Z_{\text{ip}}.$$

Here *δ*_0_ is an intercept term and *δ*_*p*_ is the effect of covariate *p* on the class membership probability.

### Estimation techniques

Both the BM and LCBM can be estimated with the Latent GOLD 4.5 software package for latent class analysis [30]. To find the Maximum Likelihood estimates for the model parameters, a combination an EM and a Newton-Raphson algorithm is used; that is, the estimation process starts with a number of EM iterations and when close enough to the final solution, the program switches to Newton-Raphson. A well-known problem in LC analysis is the occurrence of local maxima. To prevent ending up with a local solution, multiple sets of starting values are used. The user can specify the number of start sets and the number of EM iterations to be performed per set.

### Simulation study

The performance of the LCBM was evaluated using a simulation study. More specifically, we evaluated Bias (difference between the mean estimate and the true value of the parameter for both LCBM and BM), Power (the proportion of replications in which the LCBM is preferred over the BM when data are generated from a true latent class structure), and Confidence (the proportion of replications in which the BM is preferred over the LCBM when data are generated from a single class structure). We were interested in the effects of the number of studies included in the meta-analysis, within-study sample size, and true mean sensitivity and specificity on the performance of the BM and the LCBM.

The settings used in our study were taken from the simulation study by Hamza et al. [18]. The disease prevalence was fixed to 50%. Four conditions were considered for the number of studies included in the meta-analysis; that is, 10, 25, 50, and 100 studies. The study size was generated from a normal distribution and rounded to the nearest integer. Two different distributions were considered, *N*(40,30^2^) and *N*(500,450^2^). The minimum study size was set to be 10, meaning that if the generated study size was less than 10, it was set to 10. Consequently, 40 and 500 are no longer the means for the simulated study sizes, but the medians, and the realized standard deviations will be slightly smaller than 30 and 450, respectively.

For assessing the Power and the Bias, we considered scenarios with data coming from two equal size latent classes with varying patterns of sensitivities and specificities. Due to the need of simplifying the evaluation of the results, both in the first and in the second latent class the negative correlation across studies between sensitivity and specificity was kept fixed at -0.75. We considered six different patterns for the class-specific specificities and sensitivities, which crossed with the 4 sample size and the 2 within-study sample conditions, yields a total of 48 scenarios. An overview of the simulated scenarios is given in Table 1.

**Table 1:** The different scenarios used in the simulation study for assessing power

	**Class1**		**Class2**	
	**Sensitivity**	**Specificity**	**Sensitivity**	**Specificity**
1-8	90%	75%	75%	75%
9-16	90%	75%	90%	90%
17-24	90%	75%	75%	90%
25-32	90%	60%	60%	60%
33-40	90%	60%	90%	90%
41-48	90%	60%	60%	90%

Each subset of eigth scenarios corresponds to 10, 25, 50, and 100 studies in the meta-analysis and different median within-study sample size (40 and 500).

For assessing the Confidence of LCBM, we considered a set of scenarios with data coming from a single latent class, with a -0.75 correlation between sensitivity and specificity across studies. We considered three different patterns for specificity and sensitivity, which crossed with the 4 sample size and the 2 within-study sample conditions, yields a total of 24 scenarios (see Table 2).

**Table 2:** The different scenarios used in the simulation study for assessing confidence

	**Sensitivity**	**Specificity**
1-8	90%	60%
9-16	90%	75%
17-24	90%	90%

Each subset of eigth scenarios corresponds to 10, 25, 50, and 100 studies in the meta-analysis and different median study size (40 and 500).

Each scenario was replicated 1,000 times, and the simulated data sets were analyzed according to the BM and LCBM. Power and Confidence were assessed for different model selection criteria: AIC, AIC3 and BIC. These information criteria are defined as follows:

(12) $$\begin{array}{l} \text{BIC}=-2\text{LL}+\text{log}(N)\cdot \text{npar}\\ \text{AIC}3=-2\text{LL}+3\cdot \text{npar}\\ \text{AIC}=-2\text{LL}+2\cdot \mathrm{npar.} \end{array}$$

Note that the criteria differ with respect to the weighting of parsimony in terms of number of parameters. Because the log of the sample size is usually larger than 3, BIC tends to select a model with fewer latent classes than AIC and AIC3. AIC has been shown to be a superior fit-index when dealing with complex models combined with small sample sizes [31].

## Results

### Simulation study

#### Bias

**Figure 1:**
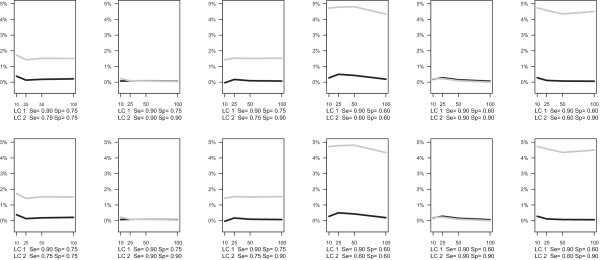
**Simulation results for median within-study sample size equals to 40.** Bias of sensitivity (top panel) and specificity (bottom panel) in LCBM (black line) and BM (grey line).

**Figure 2:**
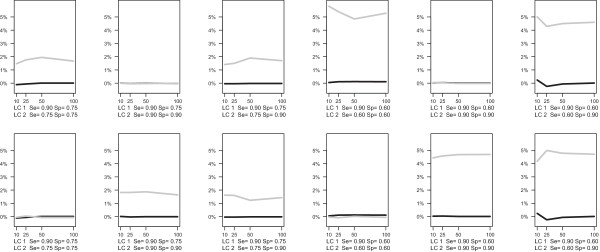
**Simulation results for median within-study sample size equals to 500.** Bias of sensitivity (top panel) and specificity (bottom panel) in LCBM (black line) and BM (grey line).

The results of the simulation study show that BM yields a systematic bias if sensitivity or specificity differs across latent classes. The BM tends to overestimate sensitivity/specifity more when the difference between the two latent class increases. For the condition with a median study size of 40 (Figure 1), the largest bias in the sensitivity is 4.8%, which occurs when the true sensitivity is 90% in one class and 60% in the other one and when the number of studies is 50. The bias of specificity reaches its maximum at 5.0% when specificity is 90% in one class and 60% in the other one and the number of studies is 25. When the median study size is 500 (Figure 2), the bias of sensitivity estimate in BM reaches its maximum at 5.8% when sensitivity is 90% in one class and 60% in the other one and the number of study is 10. The bias of specificity reaches its maximum at 5.0% when specificity is 90% in one class and 60% in the other one and the number of studies is 25. The mean bias is close to zero for the LCBM in all scenarios.

#### Power

**Figure 3:**
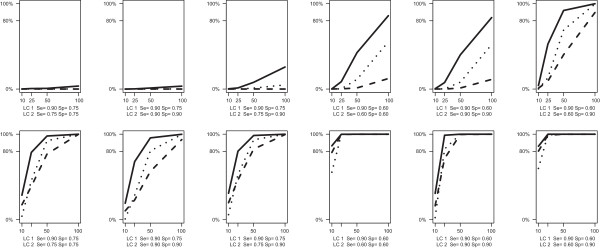
**Simulation results for median within-study sample size equals to 40 (top panel) and 500 (bottom panel).** Power of LCBM (proportion of replications in which the LCBM is preferred over the BM when data are generated following a true latent class structure) in terms of AIC (solid line) AIC3 (dotted line) and BIC (dashed line).

As we expected, when two latent class of studies exist, the probability of correctly finding this mixture (Power) goes up as we increase the number of studies, their size, and the difference in the sensitivity/specificity between the classes. Power was evaluated with AIC, AIC3 and BIC across different conditions (Figure 3). The AIC criterion is clearly preferable, because in most of the cases (except when the difference in terms of sensitivity/specificity between the classes is huge) BIC and AIC3 need more than 25 primary studies to detect the specified two-class structure.

#### Confidence

**Figure 4:**
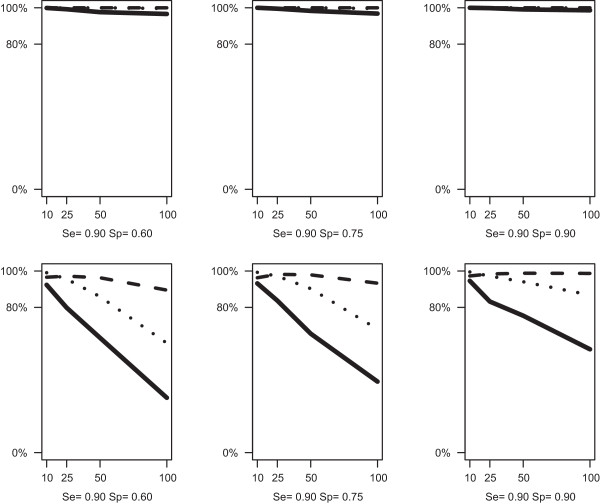
**Simulation results for median within-study size equals to 40 (top panel) and 500 (bottom panel).** Confidence of LCBM (proportion of replications in which the BM is preferred over the LCBM when data are generated within a single class structure) in terms of AIC (solid line) AIC3 (dotted line) and BIC (dashed line).

When data were simulated from a single class of studies, the probability of correctly rejecting a two component mixture (Confidence) was inversely related to the number of primary studies and their size. Confidence was also evaluated with AIC, AIC3 and BIC (Figure 4). The BIC criterion protected very well from the eventuality that LCBM finds two classes when in fact data were generated from a single class model. AIC and AIC3 were reliable criteria, up to a certain number of studies (approximately up to 25 studies for AIC and 50 for AIC3).

### Real data example: coronary artery disease data

We illustrate the use of the LCBM approach by re-analyzing data from Schuetz and others [29]. This well-known dataset is presented (Additional file 1) in the Cochrane Handbook for Systematic Reviews of Diagnostic Test Accuracy [16]. In this meta-analysis, the diagnostic performances of multislice computed tomography (CT) and magnetic resonance imaging (MRI) for the diagnosis of coronary artery disease (CAD) are compared. Prospective studies that evaluated either CT or MRI (or both), used conventional coronary angiography (CAG) as the reference standard, and used the same threshold for clinically significant coronary artery stenosis (a diameter reduction of 50% or more) were included in the review. A total of 103 studies provided a 2-by-2 table for one or both tests and were included in the meta-analysis: 84 studies evaluated only CT, 14 evaluated only MRI, and 5 evaluated both CT and MRI. Because the studies were selected based on a common threshold for clinically significant coronary artery stenosis, BM was used for data synthesis and test comparison.

Using the Latent GOLD software version 4.5 [30], we estimated both the BM and LCBM with test type as a covariate (the software code is provided in the Additional file 2). For model selection, we used the AIC. The 2-class LCBM gave a lower AIC value (AIC = 961.9) than the standard BM (AIC = 963.7), which is in fact a 1-class LCBM.

Table 3 reports the estimated sensitivities and specificities with their confidence intervals for CT and MRI studies obtained with the BM and LCBM. The LCBM identified two clusters, the first one with a sensitivity of 86.6% (95% CI = 84.5%-88.7%) and a specificity of 69.1% (95% CI = 61.8%-76.4%), and the second with a sensitivity of 97.2% (95% CI = 96.3%-98.1%) and a specificity of 84.9% (95% CI = 82.7%-87.0%). Thus we have a clear separation in the ROC space between overperforming (higher sensitivity and specificity) and underperforming studies (lower sensitivity and specificity). CT studies are mostly classified, with a probability of 85.5% (95% CI = 75.4%-95.5%), in the first latent class of (overperforming studies), and show estimated sensitivity and specificity respectively of 95.7% (95% CI = 94.6%-96.8%) and 82.6% (95% CI = 80.1%-85.0%). MRI studies are mostly classified in the second latent class (overperforming studies), with a probability of 97.5% (95% CI = 87.9%-100.0%), and have an estimated sensitivity and specificity of 86.9% (95% CI = 84.7%-89.1%) and 69.5% (95% CI = 62.2%-76.7%).

**Table 3:** Coronary hearth disease data: point estimates and confidence intervals of sensitivity and specificity in BM and LCBM both for CT and MRI studies

		**CT**	**MRI**
BM	Sensitivity	95.0% (94.0%-96.0%)	86.2% (81.4%-91.0%)
	Specificity	82.4% (80.4%-84.4%)	71.0% (64.5%-77.6%)
LCBM	Sensitivity	95.7% (94.6%-96.8%)	86.9% (84.7%-89.1%)
	Specificity	82.6% (80.1%-85.0%)	69.5% (62.2%-76.7%)

Looking at the model estimates (Table 3), we notice LCBM yields slightly different confidence intervals for sensitivity and specificity in MRI than BM.

**Figure 5:**
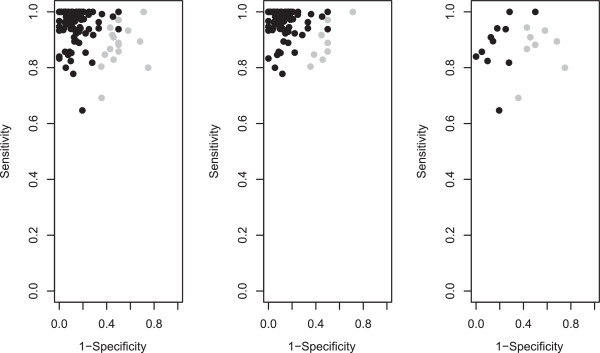
**Scatter plot in a ROC space of all (left panel), CT (middle panel) and MRI (rigth panel).** Class 1 studies in black and Class 2 studies in grey.

The obtained classification of the studies in two clusters is very clear and the ROC space is well separated (Figure 5). Classification probabilities for each study are presented, with their 95% confidence intervals in the Additional file 3. As a next step, we can investigate why a particular study is classified in the second class. It turn out that underperforming primary studies are older (38% were conducted before 2006 vs 18% in overperforming) and more often included one direct comparison study (7% vs 5% in overperforming). The class with underperforming studies could be investigate more in depth by considering other study-specific variables.

## Discussion

In the simulation study we have seen that when sensitivity or specificity differs between latent classes, BM leads to biased estimates of sensitivity and specificity. In the real data example, we obtained slightly different confidence intervals for sensitivity and specificity in MRI with LCBM.

The disadvantage of using the LCBM is the considerable increase in the number of parameters to estimate compared to the BM, implying that the number of primary studies available may become an issue. As we can see from the results of the simulation study, even when there is a strong latent class structure, to obtain a reasonable power, the number of primary studies need to be about 25. While the AIC fit criterion has to be preferred in order to achieve a reasonable power, it can lead to false positive identification of latent classes. However, false positive identification of clusters by LCBM will not end-up in biased estimates but in inflated standard errors.

In the reported simulation study, we fixed the disease prevalence to 50%. However, prevalence can take on quite different values in diagnostic accuracy studies [32]. We also simulated scenarios with a lower disease prevalence, which had little impact on the results (data not shown).

Meta-analysis of diagnostic studies with bivariate mixed-effects models can sometimes end up with non-convergence. In the LCBM, an additional discrete latent variable is added to the standard BM, which increases number of parameters and makes the computational aspects even more challenging. However, the implementation of BM and LCBM in the Latent GOLD computer program turned out to be very stable.

LCBM could be programmed in R or implemented in a SAS macro by applying PROC NLMIXED. However, the computational approach used in Latent GOLD is very stable and offers good performance in terms finding the maximum likelihood solution and convergence. Additional advantages of the Latent GOLD implementation include that it allows expanding the model in various possible ways and that several useful outputs are readily available, which would be quite complicated to program from scratch in other languages.

## Conclusions

The proposed LCBM framework provides us with a tool for assessing whether the study heterogeneity can be explained in a more careful way. It yields a clustering of studies in diagnostic test accuracy reviews that can be used for explanatory purposes.

In the real data example, we saw how LCBM can improve the understanding of the relationship between sensitivity and specificity. The LCBM is able to identify subgroups of studies that are separated in ROC space. What is added by the LCBM framework is that it provides an explanatory and confirmatory tool for investigating and testing different patterns of performance across studies. In particular, in the real data example, we tested the equivalence in diagnostic performance between CT and MRI. Moving from BM to LCBM we obtained a clear picture of two clusters of studies, and obtained more reliable confidence intervals for the sensitivity and specificity.

We can conclude that the LCBM yields a statistically rigorous, flexible, and data-driven approach to meta-analysis. LCBM generates useful results when subgroups of studies can be related to meaningful design or clinical characteristics and it provides us with a model-based starting point for subgroup meta-analyses.

Future work will include the implementation of the LCBM in an R package, the Bayesian estimation and testing of the model, and the investigation of its application in Hierarchical Summary ROC.

## Competing interests

The authors declare that they have no competing interests.

## Authors’ contributions

PE conceived research questions, developed study design and methods, carried out statistical analysis, interpreted results and drafted the manuscript. JBR advised on the study design, methods, statistical analyses and manuscript. JKV advised on the statistical analyses and software code. All authors commented on successive drafts, and read and approved the final manuscript.

## Pre-publication history

The pre-publication history for this paper can be accessed here:

http://www.biomedcentral.com/1471-2288/14/88/prepub

## Supplementary Material

Additional file 1 CAD data. Click here for file

Additional file 2 Software code for estimating BM and LCBM. Click here for file

Additional file 3 CAD posterior classification probabilities and 95% CI. Click here for file
