# Supplementary material for: Latent class bivariate model for the meta-analysis of diagnostic test accuracy studies
Source: BMC Med Res Methodol. 2014 Jul 11;14:88. doi: 10.1186/1471-2288-14-88 (PMC4105799; doi:10.1186/1471-2288-14-88)
Supplement: Additional file 2 — Software code for estimating BM and LCBM. [file 1471-2288-14-88-S2.pdf]

The model discussed in this article can be defined using LATENT GOLD software 4.5 (Vermunt JK, Magidson J. LATENT GOLD User's Manual. Statistical Innovations Inc: Boston, 2000), a latent class software that finds the ML estimates through the EM and Newton–Raphson algorithms. What we need is to define in the syntax module a series of regression equations for the latent and/or the response variables, and settings for the variances and covariances.

Before defining the regression equations, we have to specify the technical and output options, as well as the names and scale types of the latent, dependent variables that play a role in the model. The model definition for the specification of the BM and LCBM consists of simple commands on the syntax module of the software.

We specify the initial set of parameters, although local maxima and lack of convergence are avoided with the reported algorithm options.

```
//Bivariate Model
model
title 'BM test';
options
   algorithm
     tolerance=1e-008 emtolerance=0.01 emiterations=500 nriterations=50;
   startvalues
     seed=0 sets=50 tolerance=1e-005 iterations=150;
   bayes
     categorical=1 variances=1 latent=1 poisson=1;
   quadrature
     nodes=10;
   output
     parameters=effect standarderrors profile estimatedvalues
     iterationdetails;
variables
   dependent tp binomial exposure=disease, tn binomial exposure=healthy, test;
   latent t nominal 2, mu continuous, vu continuous;

equations
   t <- (0.772188) 1;
   test <- (d~wei) t;
   eta <- t;
   vu <- t;
   tp <- 1 + (1) mu;  // regression equation for sensitivity
   tn <- 1 + (1) vu;  // regression equation for specificity
   mu;                     // variance mu
   vu;                     // variance vu
   mu <-> vu;              // covariance mu-vu
   d = {1 0 0 1};

{
  0.6118007596941656       // set of initial parameters
  0.3767547470430903
  2.794938780667029
  1.453721147015295
  1.039332760965706
  0.991318408692061
  0.1325671272119704
}
end model
```

```
//Latent Class Bivariate Model
model
title 'LCBM test';
options
   algorithm
     tolerance=1e-008 emtolerance=0.01 emiterations=500 nriterations=50;
   startvalues
     seed=0 sets=50 tolerance=1e-005 iterations=150;
   bayes
     categorical=1 variances=1 latent=1 poisson=1;
   quadrature
     nodes=10;
   output
     parameters=effect standarderrors profile estimatedvalues iterationdetails;
variables
   dependent tp binomial exposure=disease, tn binomial exposure=healthy, test;
   latent t nominal 2, c nominal 2, mu continuous, vu continuous;
equations
   t <- (0.772188) 1;
   test <- (d~wei) t;
   c <- 1 + t;
   tp <- 1 | c + (1) mu;   // regression equation for sensitivity
   tn <- 1 | c + (1) vu;   // regression equation for specificity
   mu | c;            // variance mu
   vu | c;            // variance vu
   mu <-> vu | c;          // covariance mu-vu

   d = {1 0 0 1};
{
  0.4735558360355783       // set of initial parameters
  -1.358539194847309
  1.872369397280165
  3.831519465879959
  0.9143078109545019
  2.038997362223336
  0.1179006113871694
  0.7726622307426895
  1.268099770282355e-015
  0.9852456523927696
  -0.8075096254972372
  -0.2056679110205493
}
end model
```
